# Supplementary material for: AcrIIA5 Suppresses Base Editors and Reduces Their Off-Target Effects
Source: Cells. 2020 Jul 27;9(8):1786. doi: 10.3390/cells9081786 (PMC7463901; doi:10.3390/cells9081786)
Supplement: Supplementary file 1 [file cells-09-01786-s001.zip › Supplementary Table.docx]

Table S1: Target sites used for base-editing analysis.

| Target site | Genomic locus | Protospacer and PAM sequence |
| --- | --- | --- |
| site 1 | *DMD*-exon 12 | ACGCC_5_AAGTACAACAACATAAGG |
| site 2 | *DMD*-exon 10 | AGAC_4_C_5_AGTTTCATACTCATGAGG |
| site 3 | *DMD*-exon 20 | CAGAAC_6_AACTGAACAGCCGGTGG |
| site 4 | *FBN1*-exon 4 | GGTC_4_AGATAGCTCCTTCCTGTGG |
| site 5 | *FBN1*-exon 6 | AATC_4_GATGTGCATGCACTTACGG |
| site 6 | *GAPDH*-exon 6 | TTTGC_5_AGGGGGGAGCCAAAAGGG |
| site 7 | *GAPDH*-exon 8 | GCCAC_5_C_6_C_7_AGAAGACTGTGGATGG |
| site 8 | *DNMT1* | AGACCA_6_TCAGGCATTCTACCAGG |
| site 9 | *DNMT1* | TACTA_5_CTCAGCCACCAAGAACGG |
| site 10 | HEK site 4 | GATGAC_6_AGGCAGGGGCACCGCGG |
| site 11 | *FANCF* | GCGGTC_6_TC_8_AAGCACTACCTACGT |

Table S2: Primers used for base-editing analysis.

| Site | Primer sequence |
| --- | --- |
| *DMD*-exon 12 fwd | AGAGTTGAATGACTGGCTAACA |
| *DMD*-exon 12 rev | TGGTGGAGGGTCAAGAGATA |
| *DMD*-exon 10 fwd | TTGGAAGCTCCTGAAGACAAG |
| *DMD*-exon 10 rev | TTGGCCGATCAGGTAGAAATG |
| *DMD*-exon 20 fwd | TGTTGTGACGCAAGTCTGATAA |
| *DMD*-exon 20 rev | GCTCCAAATGGAAGGAGAAGAG |
| *FBN1*-exon 4 fwd | GAACCACCGTGACTGGATAAA |
| *FBN1*-exon 4 rev | GGAAATGAGAGGCCAGATGAA |
| *FBN1*-exon 6 fwd | CGGAATGAGACCATCAGCATTA |
| *FBN1*-exon 6 rev | GGGACCTTCCCAATGACAAA |
| *GAPDH*-exon 6 fwd | GGAGTGAGTGGAAGACAGAATG |
| *GAPDH*-exon 6 rev | CCTTCCTCACCTGATGATCTTG |
| *GAPDH*-exon 8 fwd | CGTGGAAGGACTCATGGTATG |
| *GAPDH*-exon 8 rev | CTGCTTCACCACCTTCTTGA |
| *DNMT1-*site 8 fwd | ACCAGAGTGAAGACTGGAGA |
| *DNMT1-*site 8 rev | CAATTTGCTCTGCGAAGAAAGTA |
| *DNMT1-*site 9 fwd | GCCTTATGTTTCTGTCCCTTTG |
| *DNMT1-*site 9 rev | CACTTACTTGAACGTGAAGGC |
| HEK site 4 fwd | GAACCCAGGTAGCCAGAGAC |
| HEK site 4 rev | TCCTTTCAACCCGAACGGAG |
| *FANCF* fwd | CAGTATGTCTCTGGCGTTACTT |
| *FANCF* rev | CCTGGAGATTTGGGTTCTCTC |
| *EMX1* on-target fwd | GCCTCCTGAGTTTCTCATCTG |
| *EMX1* on-target rev | CTAGTCATTGGAGGTGACATCG |
| *EMX1* off-target fwd | TGGGCGAGAAAGGTAACTTATG |
| *EMX1* off-target rev | TGTACTCAAGGTAAGCCTCATTATC |
| *TYRO3* on-target fwd | GGACCCTCAGAGAGACTCAA |
| *TYRO3* on-target rev | GGAGAGCCTACCTGAACTGTA |
| *TYRO3* off-target fwd | GGCACAAGGACCACAACA |
| *TYRO3* off-target rev | AAATGCCTGGCCTCTTCTC |
| *FANCF* on-target fwd | AGTTGCCCAGAGTCAAGGAACACGG |
| *FANCF* on-target rev | GACGTAGGTAGTGCTTGAGACCGCC |
| *FANCF* off-target fwd | CACTGAAGAAGCAGGGCCACACC |
| *FANCF* off-target rev | CACTGGGTGCTTAATCCGGCTCC |
| *HBG* on-target fwd | GTGGAGTTTAGCCAGGGACC |
| *HBG* on-target rev | TGGTGGGAGAAGAAAACTAGC |
| *HBG* off-target fwd | AAATGCTTCTCGGGCTCTCC |
| *HBG* off-target rev | ATGGCTGCAAATCCAAGGGT |

Table S3: DNA sequence at a cleavage site

| Genomic locus | | Sequence |
| --- | --- | --- |
| *EMX1* | On-target | GAGTCCGAGCAGAAGAAGAAGGG |
|  | Off-target | GAGTCtaAGCAGAAGAAGAAGAG |
| *TYRO3* | On-target | GGCCACACTAGCGTTGCTGCTGG |
|  | Off-target | GGCCACACTAGtGTTGCcGCTGG |
| *FANCF* | On-target | GGAATCCCTTCTGCAGCACCTGG |
|  | Off-target | GGAAcCCCgTCTGCAGCACCAGG |
| *HBG* | On-target | GTGGGGAAGGGGCCCCCAAGAGG |
|  | Off-target | GgtGGGAtGGGGtCCCCAAGTGG |

Table S4: Human codon optimized anti-CRISPR sequence

| Anti-CRISPR | DNA sequence |
| --- | --- |
| AcrIIA5 | ATGGCCTACGGCAAGTCCCGGTACAACTCTTATAGGAAGCGCAGCTTCAACAGATCCAATAAGCAGCGGAGAGAGTACGCCCAGGAGATGGATCGGCTGGAGAAGGCCTTCGAGAATCTGGACGGCTGGTATCTGAGCTCCATGAAGGACTCTGCCTACAAGGATTTTGGCAAGTATGAGATCAGGCTGTCTAACCACAGCGCCGACAATAAGTACCACGATCTGGAGAACGGCCGCCTGATCGTGAATATCAAGGCCTCCAAGCTGAACTTTGTGGACATCATCGAGAATAAGCTGGATAAGATCATCGAGAAGATCGACAAGCTGGACCTGGATAAGTATCGGTTCATCAACGCCACCAATCTGGAGCACGATATCAAGTGCTACTATAAGGGCTTTAAGACAAAGAAGGAAGTGATCTGA |
| AcrIIC1 | ATGGCCAACAAGACCTACAAGATCGGCAAGAATGCCGGCTATGACGGATGCGGACTGTGCCTGGCAGCAATCTCTGAGAACGAGGCCATCAAGGTGAAGTACCTGCGGGATATCTGCCCCGACTATGATGGCGACGATAAGGCAGAGGACTGGCTGAGATGGGGAACCGATAGCAGAGTGAAGGCAGCCGCCCTGGAGATGGAGCAGTACGCCTATACAAGCGTGGGCATGGCCTCCTGTTGGGAGTTCGTGGAGCTGTGA |
| AcrIIC2 | ATGGCCTCTAAGAACAATATCTTCAACAAGTACCCAACCATCATCCACGGAGAGGCAAGGGGAGAGAATGACGAGTTCGTGGTGCACACCAGGTATCCCCGCTTTCTGGCCAGAAAGAGCTTCGACGATAACTTTACAGGCGAGATGCCCGCCAAGCCTGTGAATGGAGAGCTGGGACAGATCGGAGAGCCTCGGAGACTGGCCTACGATAGCAGGCTGGGCCTGTGGCTGTCCGACTTTATCATGCTGGATAACAATAAGCCCAAGAACATGGAGGACTGGCTGGGACAGCTGAAGGCAGCATGCGATAGAATCGCCGCCGACGATCTGATGCTGAATGAGGACGCAGCAGATCTGGAGGGATGGGACGATTGA |
| AcrIIC3 | ATGGCCTTTAAGAGGGCCATCATCTTCACCTCTTTTAACGGCTTCGAGAAGGTGAGCCGCACAGAGAAGCGGAGACTGGCCAAGATCATCAATGCCCGGGTGTCCATCATCGACGAGTACCTGAGAGCCAAGGACACCAACGCCTCTCTGGATGGCCAGTATCGGGCCTTCCTGTTTAATGATGAGAGCCCCGCCATGACCGAGTTTCTGGCCAAGCTGAAGGCCTTCGCCGAGAGCTGCACAGGCATCTCCATCGACGCCTGGGAGATCGAGGAGTCCGAGTACGTGCGGCTGCCTGTGGAGAGGCGCGATTTTCTGGCCGCCGCCAACGGCAAGGAGATCTTCAAGATCTGA |
