# Supplementary material for: AcrIIA5 Suppresses Base Editors and Reduces Their Off-Target Effects
Source: Cells. 2020 Jul 27;9(8):1786. doi: 10.3390/cells9081786 (PMC7463901; doi:10.3390/cells9081786)
Supplement: Supplementary file 1 [file cells-09-01786-s001.zip › Supplementary Figs.docx]

Figure S1. Sanger sequencing chromatograms of cells transfected with BE3 or ABE7.10. The shadows in the Sanger sequencing results represent the sgRNA targeting sites and the three bases after the shade represent the PAM sequences.

Figure S2. Base editing quantification data of Figure S1 Sanger sequencing.

Figure S3. Sanger sequencing chromatograms of cells transfected with the BE3 and anti-CRISPR plasmids, compared with chromatograms of control cells. The shadows in the Sanger sequencing results represent the sgRNA targeting sites and the three bases after the shade represent the PAM sequences.

Figure S4. Base editing quantification data of Figure S3 Sanger sequencing.

Figure S5. Sanger sequencing chromatograms of cells transfected with the ABE7.10 and anti-CRISPR plasmids, compared with chromatograms of control cells. The shadows in the Sanger sequencing results represent the sgRNA targeting sites and the three bases after the shade represent the PAM sequences.

Figure S6. Base editing quantification data of Figure S5 Sanger sequencing.

Figure S7. Sanger sequencing chromatograms of cells transfected with the BE3 plasmid and increasing amounts of the anti-CRISPR plasmid from 1:1 to 1:5. The shadows in the Sanger sequencing results represent the sgRNA targeting sites and the three bases after the shade represent the PAM sequences.

Figure S8. Base editing quantification data of Figure S7 Sanger sequencing.

Figure S9. Sanger sequencing chromatograms of cells transfected with the ABE7.10 plasmid and increasing amounts of the anti-CRISPR plasmid from 1:1 to 1:5. The shadows in the Sanger sequencing results represent the sgRNA targeting sites and the three bases after the shade represent the PAM sequences.

Figure S10. Base editing quantification data of Figure S9 Sanger sequencing.

Figure S11. Sanger sequencing chromatograms of cells transfected with the AncBE4max and AcrIIA5 plasmids. The shadows in the Sanger sequencing results represent the sgRNA targeting sites and the three bases after the shade represent the PAM sequences.

Figure S12. Base editing quantification data of Figure S11 Sanger sequencing.

Figure S13. Sanger sequencing chromatograms of cells transfected with the ABEmax and AcrIIA5 plasmids. The shadows in the Sanger sequencing results represent the sgRNA targeting sites and the three bases after the shade represent the PAM sequences.

Figure S14. Base editing quantification data of Figure S13 Sanger sequencing.

Figure S15. Sanger sequencing chromatograms of cells transfected with the NG-AncBE4max (targeting the endogenous gene HEK-site 4) and AcrIIA5 plasmids. The shadows in the Sanger sequencing results represent the sgRNA targeting sites and the three bases after the shade represent the PAM sequences.

Figure S16. Base editing quantification data of Figure S15 Sanger sequencing.

Figure S17. Sanger sequencing chromatograms of cells transfected with the NG-AncBE4max (targeting the endogenous gene FANCF) and AcrIIA5 plasmids. The shadows in the Sanger sequencing results represent the sgRNA targeting sites and the three bases after the shade represent the PAM sequences.

Figure S18. Base editing quantification data of Figure S17 Sanger sequencing.

Figure S19. Sanger sequencing chromatograms of cells transfected with the xCas9-AncBE4max (targeting the endogenous gene HEK-site 4) and AcrIIA5 plasmids. The shadows in the Sanger sequencing results represent the sgRNA targeting sites and the three bases after the shade represent the PAM sequences.

Figure S20. Base editing quantification data of Figure S19 Sanger sequencing.

Figure S21. Sanger sequencing chromatograms of cells transfected with the CRISPR/xCas9-AncBE4max (targeting the endogenous gene FANCF) and AcrIIA5 plasmids. The shadows in the Sanger sequencing results represent the sgRNA targeting sites and the three bases after the shade represent the PAM sequences.

Figure S22. Base editing quantification data of Figure S21 Sanger sequencing.

Figure S23. Sanger sequencing chromatograms of cells transfected with the CRISPR/NG-ABEmax and anti-CRISPR plasmids. The shadows in the Sanger sequencing results represent the sgRNA targeting sites and the three bases after the shade represent the PAM sequences.

Figure S24. Base editing quantification data of Figure S23 Sanger sequencing.

Figure S25. Sanger sequencing chromatograms of cells transfected with the CRISPR/xCas9-ABEmax and anti-CRISPR plasmids. The shadows in the Sanger sequencing results represent the sgRNA targeting sites and the three bases after the shade represent the PAM sequences.

Figure S26. Base editing quantification data of Figure S25 Sanger sequencing.

Figure S27. Base editing frequencies at the *EMX1, FANCF* (**a**), *TYRO3, HBG* (**b**) on- and off-target sites in HEK293T cells were measured by targeted deep sequencing.

**Figure S28. Proper timing of AcrIIA5 delivery diminishes off-target editing events while partly sacrificing on-target editing effects. a, b** Fold inhibition of editing by AcrIIA5 at on- and off-target sites of *EMX1*, *FANCF* (**a**) and *TYRO3*, *HBG* (**b**).
